# Case Report: Congenital hepatic hemangioma with arteriovenous fistula: 2-year multidisciplinary management and outcomes

**DOI:** 10.3389/fped.2025.1511892

**Published:** 2025-04-11

**Authors:** Renping Mao, Weiwei Ruan, Jianming Zhu, Li Li, Haiyan Jiang, Yanhong Li

**Affiliations:** ^1^ Department of Neonatal Intensive Care Unit, Affiliated Women and Children’s Hospital of Ningbo University, Ningbo, China; ^2^ Department of Pediatric Surgery, Affiliated Women and Children’s Hospital of Ningbo University, Ningbo, China

**Keywords:** hepatic hemangioma, hepatic arteriovenous fistulas, encephalomalacia, neonate, rehabilitation

## Abstract

**Background:**

Congenital hepatic hemangioma with arteriovenous fistula (HHAVF) is a rare condition in newborns that may manifest as respiratory distress, pulmonary hypertension, and heart failure shortly after birth. This report describes a case of HHAVF complicated by encephalomalacia identified after transcatheter arterial embolization (TAE). However, the condition improved with multidisciplinary management and long-term follow-up.

**Case presentation:**

A full-term female infant presented with a cardiac murmur and pulmonary hypertension at birth. Contrast-enhanced CT demonstrated multiple hepatic hemangiomas with high-flow arteriovenous shunting. Pulmonary hypertension resolved after TAE; however, the recurrence of hepatic hemangioma required oral propranolol therapy, which led to complete regression by 18 months of age. Postprocedural imaging identified encephalomalacia in the right frontotemporal and parietal lobes, as well as the basal ganglia, concurrent with left-limb motor impairment. Long-term rehabilitation improved left-limb function and the extent of encephalomalacia stabilized.

**Conclusions:**

Current research primarily focuses on early cardiopulmonary complications in HHAVF, while multidisciplinary management strategies and long-term outcomes, particularly neurological manifestations, are rarely reported.

## 1 Introduction

Hepatic hemangioma (HH) is the most common benign liver tumor in the neonatal period, characterized by rapid growth followed by spontaneous regression (1, 2). In contrast, congenital hepatic arteriovenous fistula (HAVF) is a rare vascular malformation in newborns, involving direct connections between hepatic arteries and veins without capillaries. HAVF is classified into congenital and acquired types, with congenital cases making up less than 10% (3). Neonatal HAVF is predominantly congenital, with low incidence but high mortality, and may present with dyspnea, congestive heart failure, pulmonary hypertension, and portal hypertension (4, 5). Congenital hepatic hemangioma with arteriovenous fistula (HHAVF) is associated with early symptoms and increased risks of heart failure and death in neonates (6). This report describes a case of HHAVF complicated by encephalomalacia identified after interventional embolization. However, the condition improved with multidisciplinary management and long-term follow-up.

## 2 Case report

### 2.1 Disease discovery and arterial embolization treatment

A full-term female infant was delivered by cesarean section due to prenatal ultrasound findings of an elevated umbilical artery systolic-diastolic ratio (S/D 4.97) and maternal high myopia. Her birth weight was 2,750 g, with Apgar scores of 9 and 10 at 1 and 5 min, respectively, without evidence of perinatal asphyxia or intrauterine infection. The neonate was admitted to the neonatal intensive care unit (NICU) due to tachypnea shortly after birth. On admission, a chest radiograph showed an enlarged cardiac silhouette and echocardiography revealed an enlarged right atrium and ventricle, high pulmonary pressure (56 mmHg), a 5.5 mm patent ductus arteriosus, and a 5.1 mm atrial septal defect. By the 10th day, despite treatment with milrinone, dopamine, fluid restriction, and diuresis, pulmonary hypertension worsened to 109 mmHg, the heart rate increased to 180 bpm with a grade 3 continuous murmur, and progressive hepatomegaly was noted.

**Figure 1:**
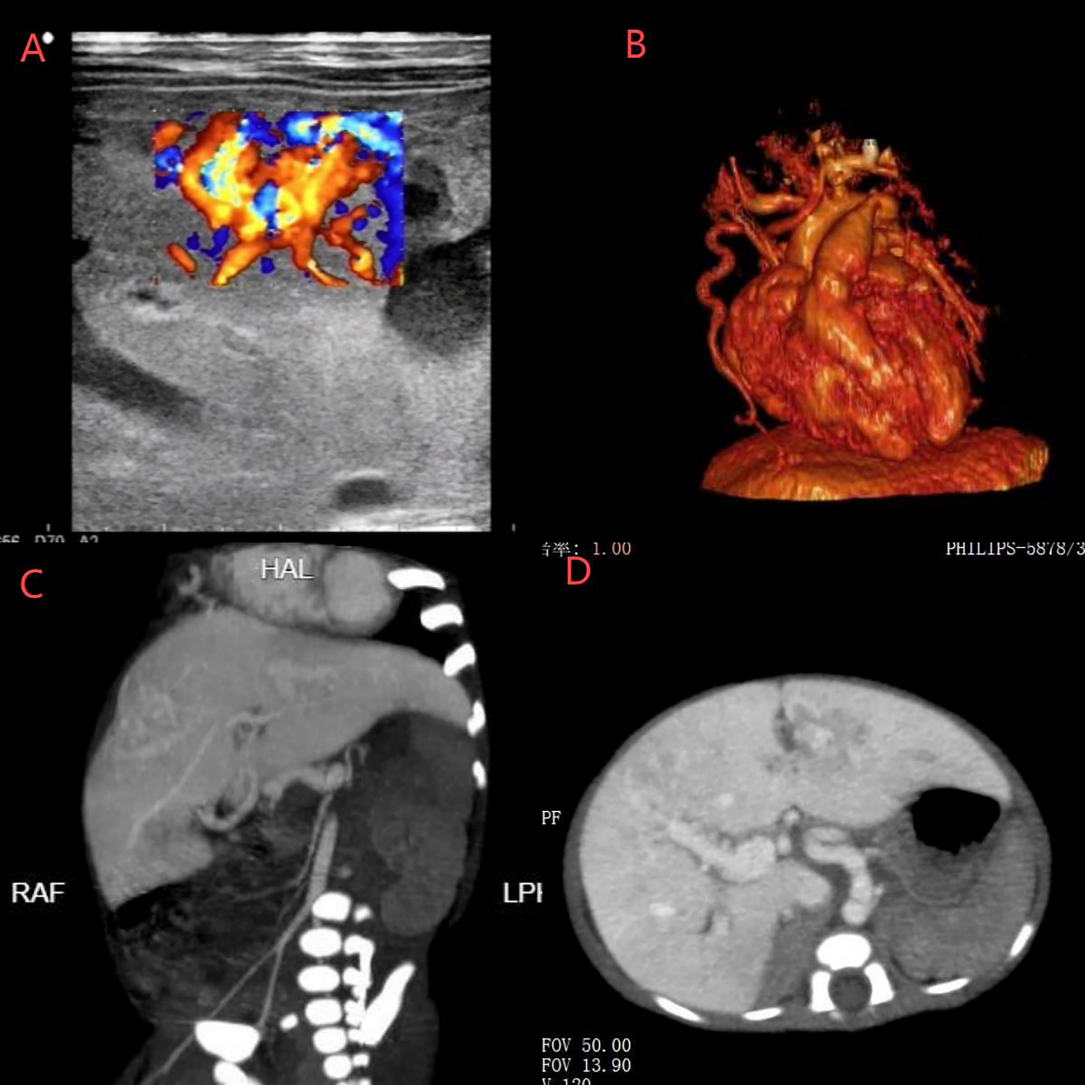
**(A)** Ultrasound showed cavernous transformation of the portal vein. **(B)** CT angiography revealed a dilated and tortuous right internal thoracic artery entering the liver. **(C,D)** CT identified hepatic artery-right portal vein fistula, hepatic portal-hepatic vein (left, middle, right) fistula, and right internal thoracic artery-left portal vein fistula.

To determine the etiology of pulmonary hypertension, comprehensive evaluations were performed. Abdominal ultrasound identified cavernous transformation of the portal vein (Figure 1A). CT angiography demonstrated a markedly dilated, tortuous right internal thoracic artery terminating in the liver, with abnormal enhancement in the left lobe and right anterior hepatic segments (Figure 1B). Contrast-enhanced abdominal CT confirmed multiple hepatic arteriovenous fistulas (Figures 1C,D). After a multidisciplinary review, transcatheter arterial embolization (TAE) using Gelfoam particles was performed via the right internal thoracic artery, proper hepatic artery, and bilateral hepatic arteries on postnatal day 20 to treat both vascular anomalies and congestive heart failure.

### 2.2 Follow-up of hepatic hemangioma with arteriovenous fistula

Within 1 week after TAE, symptoms of congestive heart failure and pulmonary hypertension improved. The right atrium and ventricle reduced in size and the ductus arteriosus closed spontaneously 2 months later. The patient subsequently required surgery to repair a persistent atrial septal defect (ASD) at the age of 2 years.

**Figure 2:**
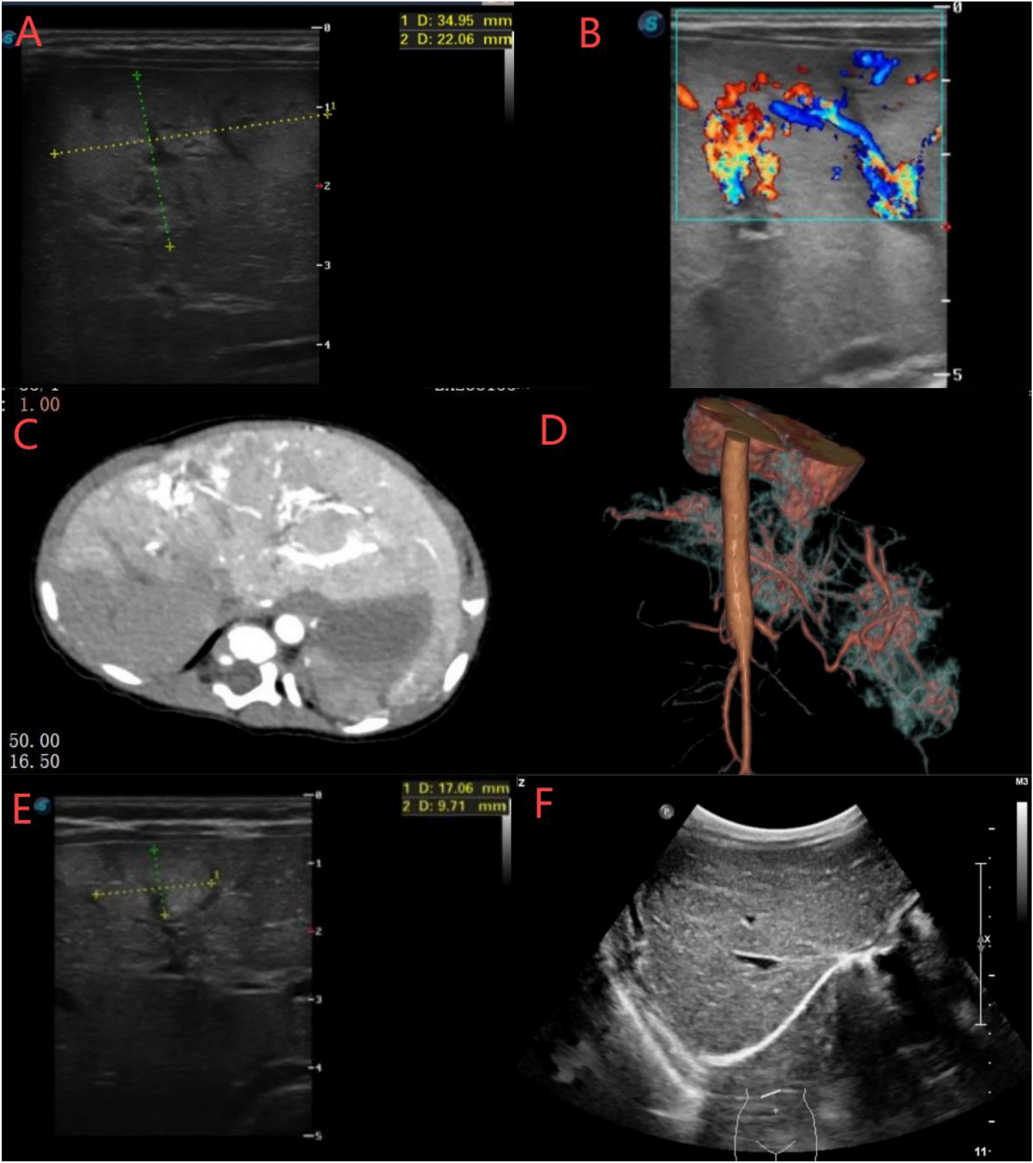
**(A,B)** Ultrasound showed multiple hyperechoic areas in the left and right hepatic lobes with cavernous echotexture and disordered vascular proliferation. **(C,D)** CT revealed significant liver enhancement with four large, irregular “snowflake”-like areas containing tortuous and dilated vessels. **(E,F)** Ultrasound showed that the hepatic hemangiomas gradually decreased in size and completely disappeared at month 18.

Unfortunately, the hemangioma grew rapidly after TAE. An ultrasound performed 13 days postoperatively showed multiple hyperechoic areas in the left hepatic lobe (largest: 35 × 22 mm) with a cavernous echogenic texture and disordered vascular proliferation (Figure 2A). By postoperative day 23, hyperechoic areas were observed in both lobes, with the largest measuring 31 × 15 × 25 mm (right lobe) and 26 × 20 × 25 mm (left lobe) (Figure 2B). Contrast-enhanced CT at 30 days postoperatively revealed four areas of arterial phase heterogeneous enhancement with tortuous and enlarged blood vessels, with the largest measuring approximately 26.5 × 4 mm (Figures 2C,D). A general surgery consultation noted that the neonate had multiple hepatic hemangiomas with abundant blood supply and rapid collateral circulation developed postoperatively. Oral propranolol was initiated at 0.2 mg/kg twice daily, increasing by 0.2 mg/kg every 3 days until reaching 1.0 mg/kg twice daily. After 3 months of oral propranolol treatment, hyperechoic hepatic masses gradually decreased in size. Ultrasound revealed the largest hyperechoic area (17 × 9 mm) at 4 months postoperatively, decreasing to 11 × 6 mm by 6 months (Figure 2E). By 10 months, only scattered punctate hyperechoic foci persisted in the right hepatic lobe. Complete resolution of multiple hepatic hemangiomas was achieved at 18 months postoperatively (Figure 2F). Subsequently, propranolol was tapered and discontinued over a 6-month period, with no recurrence of vascular lesions observed during follow-up.

### 2.3 Encephalomalacia discovery and follow-up

Preoperative brain MRI was not performed due to the absence of clinical neurological symptoms before the embolization procedure, as routine postnatal cranial ultrasound and neurological exams were unremarkable. However, 10 days postoperatively (day 31 of life), the infant presented with left-sided nasolabial flattening during crying, left-sided limb hypokinesia, ipsilateral hypertonia, and a positive palmar grasp reflex.

**Figure 3:**
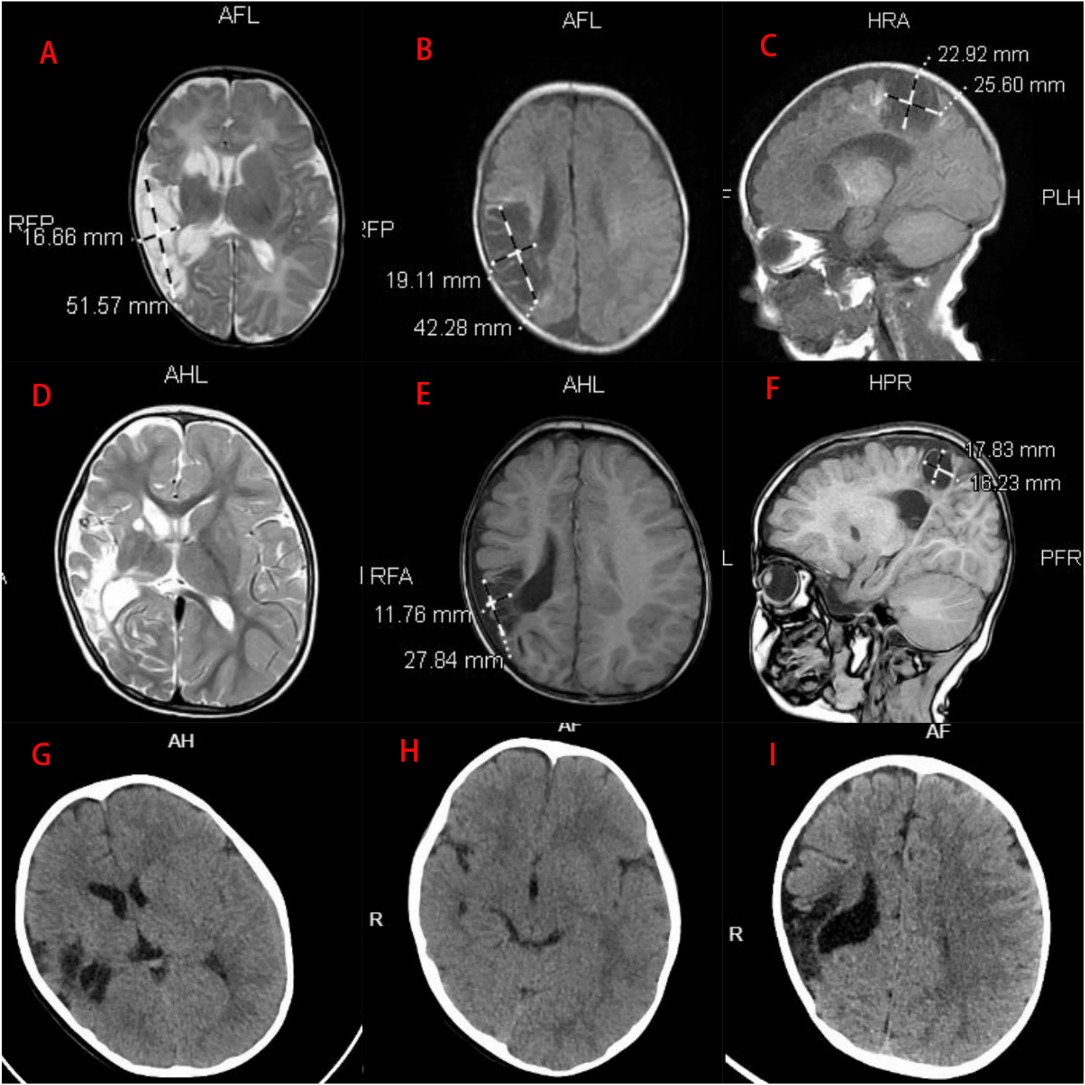
**(A–C)** Brain MRI at day 40 of life showed extensive encephalomalacia localized to the right fronto-temporal-parietal lobe and right basal ganglia (same scale). **(D–F)** Follow-up MRI at month 18 demonstrated stabilization of encephalomalacia in these regions (same scale). **(G–I)** Brain CT at month 24 revealed right hemispheric volume reduction with hypodense lesions in the right temporoparietal areas.

Meanwhile, a routine ultrasound detected a right periventricular hypoechoic lesion (20 × 12 mm). Brain MRI scans at 40 and 60 days of life showed extensive encephalomalacia in the right frontotemporal and parietal lobes and basal ganglia. The scans revealed cerebral hemisphere asymmetry, with the right hemisphere smaller in volume. Abnormal signal intensities were observed in the right frontal, parietal, and temporal lobes, as well as the basal ganglia (caudate and lentiform nuclei). T1-weighted imaging showed hypointense areas with scattered hyperintense foci, T2-weighted imaging demonstrated markedly hyperintense signals, and diffusion-weighted imaging (DWI) appeared hypointense (Figure 3A–C). Motor rehabilitation initiated at 2.5 months of age included tactile hand stimulation, active-passive limb mobilization, prone head elevation training, midline alignment, and audiovisual-language therapy. By 3.5 months, the infant exhibited full-field visual tracking (180°) and sound-localizing head turns. Prone head elevation improved to 60° for 10 s. In addition, there was an increase in social smiling and reciprocal vocalizations. However, left-sided deficits persisted, characterized by reduced spontaneous movement and ipsilateral hypertonia.

The patient received inpatient, outpatient, and home-based rehabilitation, achieving developmental milestones such as head control at 4 months, independent rolling at 6 months, bilateral palmar grasp with hand-to-hand transfer at 7 months, independent sitting at 10 months, and reduplicated babbling (e.g., “mama”) at 11 months. However, despite these achievements, Gesell Developmental Scores at 7 months revealed the following: significant delays in gross motor skills (53), personal-social skills (53), and fine motor skills (44); mild delays in adaptive behavior (54); and language (72).

By 18 months, the patient achieved independent ambulation with persistent left-sided movement restriction. Serial MRI demonstrated stabilization of right fronto-temporo-parietal and basal ganglia encephalomalacia, with progressive reduction of perilesional edema over 18 months of therapy (Figures 3D–F). At 2 years of age (weight 11 kg, height 88 cm), the child achieved independent ambulation with a stiff running pattern, no jumping ability, and an equinus gait. Gesell Developmental Scores indicated borderline gross motor (80), mild delay in fine motor (75), and normal adaptive behavior (90), language (90), and personal-social skills (89). Serial cranial CT scans revealed right hemispheric volume reduction with extensive hypodense lesions in the right temporoparietal regions (Figures 3G–I).

## 3 Discussion

HAVF is a rare congenital vascular malformation associated with high mortality. As previously reported, over 50% of HAVF patients develop heart dysfunction, with 50%–90% succumbing to severe heart failure within the first week of life (7). It is classified into three types: hepatic artery-to-portal vein, hepatic artery-to-hepatic vein, and portal vein-to-hepatic vein fistulas, each associated with distinct clinical manifestations (3, 8). Our patient presented with concurrent hepatic artery–right portal vein and hepatic portal–vein fistulas, with the left portal vein supplied by the right internal thoracic artery. Multiple abnormal arteriovenous shunts between the hepatic artery, portal vein, and hepatic vein, combined with a large hepatic hemangioma, led to increased right cardiac load and pulmonary congestion. This resulted in severe pulmonary hypertension, right atrial and ventricular enlargement, and postnatal tachycardia (9).

Ultrasound, CT, and angiography are essential diagnostic modalities for HHAVF. Diagnosis is typically achieved by integrating clinical presentation with imaging findings (3, 10). Current therapeutic approaches primarily involve surgical resection, interventional procedures, and pharmacotherapy. However, surgical intervention is generally not recommended for neonates with congestive heart failure due to poor tolerance and high risks of hemorrhage-related complications.

Compared to surgical intervention, TAE has emerged as the preferred approach due to its minimally invasive nature, reduced perioperative stress, and ability to rapidly block arteriovenous shunting while alleviating symptoms (11, 12). After TAE, the infant showed no recurrence of dyspnea, heart failure, or pulmonary hypertension, confirming its therapeutic efficacy. However, neonatal TAE remains technically challenging, particularly in cases of congenital hepatic hemangiomas with arteriovenous fistulae (9). Prognostic variability in these benign tumors correlates with tumor size, growth rate, and intralesional shunting, manifesting clinically from asymptomatic to life-threatening presentations (13). In this case, rapid hemangioma proliferation occurred after TAE due to collateral circulation. Propranolol reduces lesion size by vasoconstriction and inhibition of vascular endothelial growth factor and fibroblast growth factor generation, as well as cell proliferation. Low-dose oral propranolol (1–2 mg/kg/day) demonstrated efficacy in treating infantile hemangiomas (6, 13). In this case, the hepatic hemangiomas resolved completely after 18 months of follow-up, with no recurrence observed after gradual tapering and discontinuation of propranolol therapy.

The current literature documents early-stage HAVF with symptoms such as congestive heart failure, pulmonary hypertension, portal hypertension, and gastrointestinal bleeding (3, 8, 12). To date, no cases of HAVF associated with unilateral encephalomalacia identified after arterial embolization have been reported. Encephalomalacia involves brain tissue necrosis and liquefaction secondary to ischemia and hypoxia, leading to localized softening. It typically manifests after a latent period that can vary in duration and may result in severe neurological sequelae, including limb hypokinesia, cerebral palsy, intellectual disability, and epilepsy (14).

The right hemispheric encephalomalacia in this infant may reflect multifactorial contributions to cerebrovascular injury. An elevated umbilical artery S/D ratio (4.97) and right hemisphere volume loss could predispose the infant to cerebral vulnerability and hypoxic-ischemic injury *in utero* (15–17). However, normal birth neurological exams and cranial ultrasound argue against significant prenatal injury. Postnatal hemodynamic stressors, including congenital heart defects (PDA, ASD) and progressive pulmonary hypertension, as well as high-output heart failure from hepatic arteriovenous fistula may exacerbate systemic perfusion deficits and typically cause bilateral watershed injuries rather than unilateral focal lesions (18–20). Of note, the focal encephalomalacia involving the right fronto-temporal-parietal cortex and basal ganglia localizes to the right middle cerebral artery (MCA) territory, raising the possibility of an embolic mechanism and aligning with the characteristic pattern of embolic stroke (20, 21). Perioperative hemodynamic instability during arterial embolization, especially in high-flow arteriovenous fistula, potentially via paradoxical emboli or transient hypoperfusion, likely contributed to delayed ischemic injury (22). Furthermore, left-sided limb hypokinesia appeared 10 days after TAE whereas DWI hypointensity was observed 20 days after TAE on MRI, aligning with embolic infarct progression (21, 23). While the temporal association with TAE suggests a possible thromboembolic origin, the lack of preprocedural brain imaging necessitates cautious interpretation. Preprocedural brain MRI and intraoperative embolic monitoring should be prioritized in neonates undergoing TAE for high-flow vascular anomalies. Long-term neurodevelopmental surveillance is critical, as encephalomalacia in this region may predispose to motor deficits, as demonstrated in the current case. Although thrombi or emboli formed within placental vessels or metabolic disorders were less likely, their exclusion remains prudent (24). Genetic testing did not reveal clinically significant variants related to this child's condition, and no placental thrombi were identified on pathological examination (4).

In neonatal TAE, multimodal strategies to prevent potential perioperative emboli include: preprocedural strategies such as detailed MRI and ultrasound examinations, assessment of placental thrombosis and vascular injuries (24); intraoperative measures incorporating real-time imaging guidance (e.g., ultrasonography), perioperative anticoagulation management (e.g., low-dose heparin), and the use of miniature embolic protection devices (e.g., IVC filters) (4, 25); and postprocedural protocols must emphasize serial neurological assessments with neuroimaging surveillance (MRI-DWI) and tailored anticoagulation regimens. From our experience with this case, timely and accurate neuroimaging findings can identify infants at risk for neurodevelopmental impairment. Early and consistent rehabilitation therapy can greatly improve the prognosis of the infant (20). Boyd et al. suggested that rehabilitation therapy can be initiated as early as possible after a brain injury. As the child grows older, the duration of these exercises can be extended accordingly. Furthermore, the involvement of both parents in the child's rehabilitation treatment plays a crucial role (26).

## 4 Conclusion

Prenatal hypoxia, congenital cardiovascular anomalies, and iatrogenic interventions may be associated with severe brain injury. Perioperative hemodynamic instability during arterial embolization, potentially via paradoxical emboli or transient hypoperfusion, may represent a potential mechanism underlying delayed ischemic injury in neonates undergoing such procedures. Management strategies for this hepatic hemangioma with arteriovenous malformation encompass cardiac dysfunction therapy, arterial embolization, and pharmacotherapy. Multidisciplinary team coordination involving neonatology, cardiology, and pediatric surgery is critical to optimize clinical outcomes. Sustained neurorehabilitation remains pivotal for addressing encephalomalacia-related motor and cognitive deficits.

## Data Availability

The raw data supporting the conclusions of this article will be made available by the authors, without undue reservation.
